# An Intelligent Detector for Sensing Pork Freshness In Situ Based on a Multispectral Technique

**DOI:** 10.3390/bios12110998

**Published:** 2022-11-10

**Authors:** Wenlong Zou, Yankun Peng, Deyong Yang, Jiewen Zuo, Yang Li, Qinghui Guo

**Affiliations:** College of Engineering, National R & D Center for Agro-Processing Equipment, China Agricultural University, 17 Qinghua East Road, Haidian, Beijing 100083, China

**Keywords:** pork freshness, multi-channel spectral sensor, spectral shape feature, qualitative analysis, on-site detection

## Abstract

Fresh pork is prone to spoilage during storage, transportation, and sale, resulting in reduced freshness. The total viable count (TVC) and total volatile basic nitrogen (TVB-N) content are key indicators for evaluating the freshness of fresh pork, and when they reach unacceptable limits, this seriously threatens dietary safety. To realize the on-site, low-cost, rapid, and non-destructive testing and evaluation of fresh pork freshness, a miniaturized detector was developed based on a cost-effective multi-channel spectral sensor. The partial least squares discriminant analysis (PLS-DA) model was used to distinguish fresh meat from deteriorated meat. The detector consists of microcontroller, light source, multi-channel spectral sensor, heat-dissipation modules, display system, and battery. In this study, the multispectral data of pork samples with different freshness levels were collected by the developed detector, and its ability to distinguish pork freshness was based on different spectral shape features (SSF) (spectral ratio (SR), spectral difference (SD), and normalized spectral intensity difference (NSID)) were compared. The experimental results show that compared with the original multispectral modeling, the performance of the model based on spectral shape features is significantly improved. The model established by optimizing the spectral shape feature variables has the best performance, and the discrimination accuracy of its prediction set is 91.67%. In addition, the validation accuracy of the optimal model was 86.67%, and its sensitivity and variability were 87.50% and 85.71%, respectively. The results show that the detector developed in this study is cost-effective, compact in its structure, stable in its performance, and suitable for the on-site digital rapid non-destructive testing of freshness during the storage, transportation, and sale of fresh pork.

## 1. Introduction

Fresh pork is favored by consumers all over the world due to its freshness, tenderness, deliciousness, and nutritional content, but it is also considered to be one of the most perishable foods [1,2]. It is extremely susceptible to microbial contamination and other environmental factors in all aspects of production, and a large number of harmful substances are produced under the action of exogenous microbial reproduction and enzyme-catalyzed reactions, which seriously threatens food safety and public health [3]. Freshness is an important indicator to determine changes in the quality of fresh pork and to measure whether it is edible [4]. The total viable count (TVC) and total volatile basic nitrogen (TVB-N) content are the key indicators for freshness evaluation, and countries and organizations such as China and the FAO have clearly stipulated this. Therefore, judging the freshness of fresh pork based on TVC and TVB-N content is increasingly valued by producers and consumers. Traditional detection methods based on physicochemical and biological analyses, such as the Kjeldahl method [5] and colony counting method [6], can achieve accurate measurements, but they are time-consuming and destructive. It is thus difficult to meet the needs of producers and consumers for rapid on-site non-destructive detection. Therefore, there is an urgent need for real-time and rapid non-destructive detection technology and equipment for freshness to guide the production and processing by producers and the consumption by consumers [7,8].

Optical detection technologies, such as visible/near-infrared spectroscopy [9] and hyperspectral technology [10], have been widely used in the rapid non-destructive detection of fresh meat. In particular, near-infrared spectroscopy has been accepted by the National Standards Committee of the People’s Republic of China as the standard method for the quality inspection of livestock and poultry meat. However, most of these methods involve the use of expensive spectroscopic instruments and computers, which are bulky and are not conducive to low-cost and rapid non-destructive detection in the field [11,12]. At the same time, the spectra obtained by such methods not only contain useful spectral information, but also contain a large number of other spectral responses caused by the measured object’s own factors, such as composition, texture, and density, which affect the discrimination accuracy and efficiency of the model [13,14]. Multispectral technology is one method for reducing the cost of optical detection technology and simplifying the model, and it has been widely used in the quality detection of agricultural and sideline products [15]. Compared with other spectroscopic techniques, although the resolution of multispectral technology is limited, its cost is low, the wavelength range can be selected according to the situation, which is beneficial to the development of portable devices, and the model is more interpretable [16]. Multispectral systems with halogen lamps, filters, and photodiodes or LED light sources and photodiodes as the core components are widely used [17,18]. Based on the combination of halogen lamps, multiple optical filters, and photodiodes, Guo Wenchuan [19] developed a multispectral system to distinguish chlorfenuron-treated and untreated kiwifruit. However, the volume and cost of traditional optical filters and photodiodes restrict the choice of the number of characteristic wavelengths. Wei Wensong [20] constructed a multi-spectral detection system based on LED light sources with characteristic wavelength bands by optimizing the wavelengths related to TVB-N content in pork. Although the TVB-N content can be predicted, the light source control and arrangement of the system are complex. Detection can be completed only after LED lights of different wavelengths are turned on in sequence, and the detection efficiency is low.

With the development of micro-electromechanical processing technology and nano-optical deposition interference filter technology, chip-level spectral sensor technology has gradually matured [21,22]. A multi-channel spectral sensor can be selected, combined or even customized according to the needs of a sensor array. It has the advantages of small size, high integration, fast response, low energy consumption, good reproducibility, and easy secondary development. In this study, a multispectral system for freshness detection was established using multi-channel spectral sensors and low-power halogen lamps, and software and hardware systems were developed. At the same time, multispectral information was mined through spectral shape feature (SSF) preprocessing, and then a freshness discrimination model was established based on the total number of colonies and volatile base nitrogen content. A handheld freshness detection device for fresh pork has been developed in order to realize the fast, low-cost, on-site freshness detection of fresh pork and provide guidance for the safe production and consumption of fresh pork.

## 2. Materials and Methods

### 2.1. Fresh Pork Samples

The fresh pork samples used in the experiment were purchased from a supermarket in Beijing, and the longissimus dorsi of large white pigs that had been cooled for 24 h after slaughter were selected. After purchase, they were transported to the laboratory within one hour using a cold storage device. Under sterile conditions, the pork was cut into pieces to remove the fat and connective tissue and then divided into 5 cm × 5 cm × 2.5 cm samples with a flat surface. Each sample was individually stored in a sterilized Ziplock bag, numbered, and stored at 4 °C in a refrigerator without any backlog. A total of 96 pork samples of different freshness levels were obtained.

### 2.2. Hardware Design

**Figure 1:**
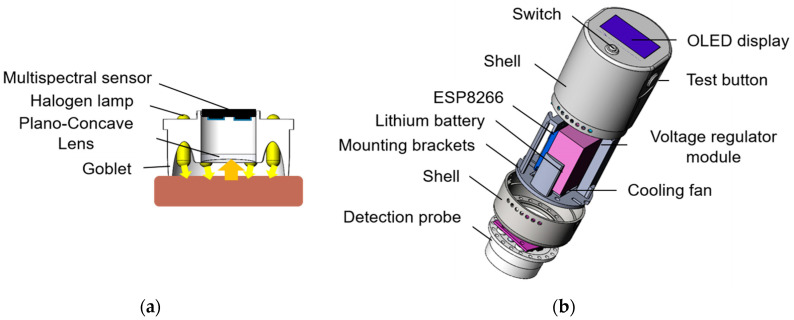
Detector structure diagram: (**a**) Schematic diagram of the detection probe; (**b**) Schematic diagram of detector structure.

Since the propagation law of the detection light in the meat sample conforms to the diffuse transmission theory, when the detection light irradiates the meat sample, it will experience absorption, scattering, and reflection inside the meat [23]. Diffuse light intensity represents the ability of meat components to interact with light. Therefore, the diffuse reflectance technique was used to collect the multispectral information of pork, as shown in Figure 1a. The detection light cup combined with the low-power halogen lamps arranged around the circumference can cause the detection probe to form a uniform annular detection light on the detection surface of the meat sample. Diffuse light from the surface of the meat sample is concentrated by the lens to a multispectral sensor arranged in the center of the detection probe where it is received. The designed handheld fresh pork freshness detector is shown in Figure 1b and mainly includes a detection probe, an ESP8266 development board, an OLED display system, a voltage regulator module, a lithium battery, a cooling fan, and a shell. When the detection system starts to work, the diffuse reflection multispectral data collected by the detection probe can be received and analyzed by the ESP8266 to obtain the detection result, which is displayed on the display screen. It can also be controlled and displayed through a remote control terminal.

The diffuse reflection detection probe, composed of a multi-channel spectral sensor and detection light source, is the core part of the detection system. Its response range covers the visible/near-infrared band, and it can output 18 bands of spectral information at the same time. The central wavelengths are 410 nm, 435 nm, 460 nm, 485 nm, 510 nm, 535 nm, 560 nm, 585 nm, 610 nm, 645 nm, 680 nm, 705 nm, 730 nm, 760 nm, 810 nm, 860 nm, 900 nm, and 940 nm, with a full width at half maximum (FWHM) of 20 nm, which has the advantages of high stability and temperature self-correction. With six 1.2 W halogen lamps (PHILIPS, 12516CP, Amsterdam, The Netherlands), it can generate a continuous visible/near-infrared spectrum, which is conducive to the simultaneous acquisition of a multi-channel spectrum. ESP8266 series modules integrate an ultra-low-power Tensilica L106 32-bit RISC processor, a storage unit, and a Wi-Fi data transceiver and other hardware on a small chip. As a microcontroller, it can perform real-time digital signal processing and low-voltage control operations. The cost is low, and it is convenient for secondary development and device function expansion, so ESP8266 is selected as the microcontroller of the detection device. The temperature control module can monitor the temperature of the device in real time and control the working state of the cooling fan according to the set temperature threshold to maintain a constant system temperature and reduce the impact of temperature changes on the system’s detection performance. The design of the system is compact, and the diameter of the detection probe is only 4 cm, which is conducive to the realization of the in situ, rapid detection and evaluation of pork freshness.

### 2.3. Software Development

**Figure 2:**
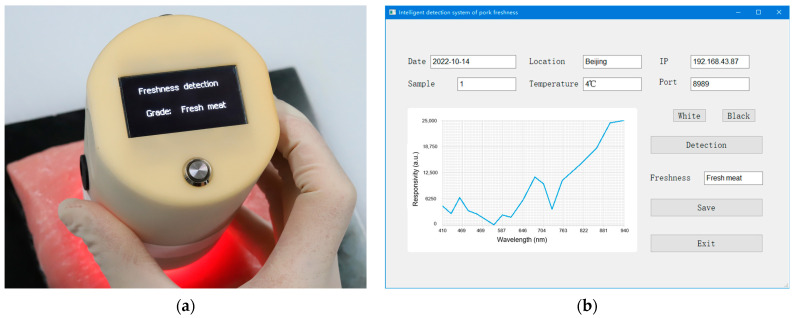
(**a**) Pork freshness detector prototype; (**b**) Pork freshness detector remote control interface.

The control software of the detector was developed using C language in the Arduino IDE development environment. The software can perform multispectral calibration, multispectral acquisition and analysis, test result display, and test data transmission and preservation. The detector can be controlled and displayed independently through the detection button, as shown in Figure 2a. It can also be controlled and displayed remotely in real time through LAN, and the remote control interface is shown in Figure 2b. The remote control interface includes a basic parameter setting and display area (date, location, IP and sample number, ambient temperature, and port), a spectrum display area, a detection control area (calibration button, collection button, and save button), and a result display area.

### 2.4. Physical and Chemical Value Determination

#### 2.4.1. TVC Physical and Chemical Value Determination

The colony culture and count of fresh meat samples were carried out according to China National Standard GB 4789.2–2016. After collecting the diffuse reflectance spectra of fresh pork samples, colony culture and counting were carried out under sterile conditions. The TVC calculation is shown in Equation (1):

(1) $$N=\frac{\sum C}{\left(n1-n2\right)d}$$

In the formula, *N* is the total number of colonies; *C* is the sum of the suitable plate colonies; *n*1 is the low dilution plate colony; *n*2 is the high dilution plate colony; and *d* is the dilution factor.

#### 2.4.2. TVB-N Physical and Chemical Value Determination

The standard value of TVB-N content in the fresh pork samples was measured according to China National Standard GB 5009.228–2016. The instruments used were mainly a KDY-9820 semi-automatic K-type nitrogen analyzer and a BL25B12 stirring cup. After collecting the diffuse reflectance spectra of the fresh pork samples, we took 10 ± 0.001 g of the homogenized samples and mixed them with 100 mL of distilled water and homogenized them for 2 min. The homogenized mixture stood for 30 min and was then filtered, and the filtered supernatant was used for subsequent titrations. The formula for calculating TVB-N content is shown in Equation (2):

(2) $$X=\frac{\left(14\times \left(V1-V2\right)\times C\right)}{\left(m\times 10/100\right)}\times 100$$

In the formula, *X* is the TVB-N content in the sample, in mg/100 g; *V*1 is the volume of standard titration hydrochloric acid consumed by the sample, in mL; *V*2 is the volume of standard titration hydrochloric acid consumed by the blank, in mL; *C* is the concentration of the standard hydrochloric acid solution, in mol/L; and *m* is the sample mass, in g.

### 2.5. Multispectral Data Acquisition and Preprocessing

The detector was preheated for at least 30 min, and then the black reference (turning off the light source) and white reference (Teflon) were collected, respectively. After the spectral calibration, the diffuse reflectance multispectral information of the meat sample could be collected. Each sample was tested five times in this study, and the average was calculated for subsequent analysis. The whole process took about five seconds. Because preprocessing methods such as multiple scattering correction (MSC) and standard normal variable transformation (SNV) usually need to operate on the continuous bands to obtain the corrected spectrum, and because the shape of the spectrum and the position of the characteristic peak or trough change easily after processing, the spectral information of single wavelength is affected [24]. Therefore, they are not suitable for the pretreatment of discrete wavelength spectral data. Spectral shape features (spectral ratio (SR), spectral difference (SD), and normalized spectral intensity difference (NSID)) were calculated without using the whole or broadband spectrum and were suitable for discrete wavelength spectral preprocessing [24,25]. Zhang Mengsheng [25] used the combination of SR, SD, and NSID to establish the apple maturity discrimination model, and the prediction accuracy was 88.46%. Moscetti [26] compared the ability of different spectral shape feature parameters to detect hazelnut kernel defects and selected the three best SR variables to establish a discriminant model with an accuracy of 94.8%. Therefore, in this paper, the spectral shape features (SR, SD, and NSID) were used to preprocess the multispectral data to improve the efficiency of the multispectral information. Spectral shape features can be calculated by three different Equations:

(3) $$R_{SR}=\frac{R_{i}}{R_{j}}$$

(4) $$R_{SD}=R_{i}-R_{j}$$

(5) $$R_{NSID}=\frac{\left(R_{i}-R_{j}\right)}{\left(R_{i}+R_{j}\right)}$$

In the formulas, *R_i_* represents the spectral reflectance of the *i*-th wavelength in the spectral curve, and *R_j_* represents the spectral reflectance of the *j*-th wavelength in the spectral curve.

### 2.6. Model Building and Evaluation

In this study, partial least squares discrimination analysis (PLS-DA) was used to discriminate between pork with different freshness levels. PLS-DA adopts the classical partial least squares regression model, and its response variable is a group of categorical information that reflects the category relationship between statistical units, which makes it a supervised discriminant analysis method [26,27]. It is often used to grade meat quality [28,29]. In PLS-DA, matrix *X (N* × *J)* is used as the input variable, and the dummy variable is *Y (N* × *1)*, such as 0, 1, and 2 are used as output variables, and its linear relationship can be described as:

(6) $$Y=Xb^{T}+e$$

In the formula, *b (*1 × *J)* is the vector of regression coefficients, *e (N* × 1*)* is the error vector, and *N* and *J* are the number of subjects (*N* is the sample size and *J* is the number of variables).

The performance of the multivariable calibration model obtained by the PLS-DA method was evaluated by sensitivity, specificity, and accuracy [30]. The calculation formulas are as follows:

(7) $$Sensitivity=\frac{TP}{TP+FN}$$

(8) $$Specikficity=\frac{TP}{TP+FP}$$

(9) $$Accuracy=\frac{\left(TP+TN\right)}{\left(TP+TN+FP+FN\right)}$$

In the formulas, *TP* represents the number of positive samples predicted as positive by the discriminant model; *TN* represents the number of negative samples predicted as negative by the discriminative model; *FP* represents the number of negative samples predicted as positive by the discriminative model; and *FN* represents the number of positive samples predicted as negative by the discriminative model.

## 3. Results

### 3.1. Physicochemical Value Statistics and Freshness Grading

**Figure 3:**
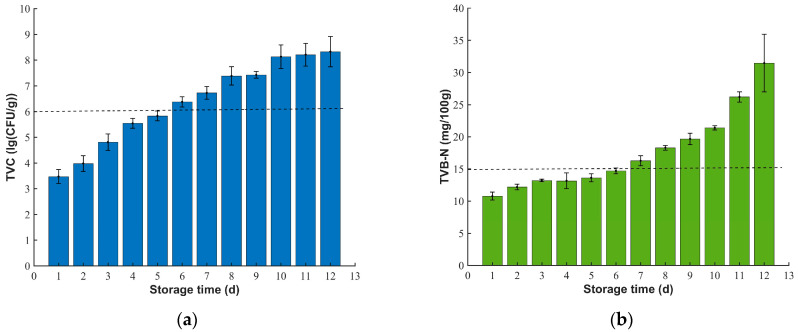
Changes in the mean values of pork freshness indicators during storage. (**a**) TVC; (**b**) TVB-N. (Standard error bars are provided, with each bar representing the mean ± standard deviation.)

We prepared and stored the samples using the method described in Section 2.1. During the experiment, every 24 h or so, eight samples were taken out from the cold room (4 °C) in sequence of sample number and left at room temperature (22–24 °C) for 30 min. Then, the diffuse reflectance multispectral spectra of the samples were collected by the designed detector. The physicochemical data of the samples were determined by the method described in Section 2.4 as soon as possible after the spectrum collection, and a total of 96 valid samples were obtained over 12 days. Figure 3 shows the changes in the TVC and TVB-N contents in the fresh pork with storage time. With the prolongation of storage time, the average value of pork TVC increased with an S-shaped trend (Figure 3a), which is similar to the change law in the published literature [4,10]. The growth of spoilage bacteria goes through a lag phase, a logarithmic phase, a stationary phase, and an extinction phase. When the internal environment of the package is suitable, the spoilage organism enters the logarithmic phase to rapidly multiply and decompose nutrients such as pork proteins and amines. During the extinction period, the total number of spoilage organisms eventually stabilizes [3,31]. However, the TVB-N content of pork increased with an increase in storage time, and the increase in speed became greater and greater. The phase of rapid increase began at around day six (Figure 3b). TVB-N is mainly produced by the degradation of pork proteins and amines by spoilage bacteria. Therefore, with an increase in TVC in the short term, the production speed of TVB-N also increases. At the same time, TVB-N may accumulate rapidly with the degradation and oxidation of amines, proteins, and lipids by endogenous enzymes. Under the action of the two, the content of TVB-N is significantly increased [32].

According to China National Standard GB 2707–2016, the TVC limit value of fresh meat is 6 lg (CFU/g), and the TVB-N content limit value is 15 mg/100 g. In this study, the TVC of pork had reached the limit value stipulated by the standard by about the fifth day, while the TVB-N content of pork did not exceed the limit value specified by the standard until the sixth day. This shows that before the TVB-N content of pork reaches the national standard limit value, the pork has already begun to spoil. When the TVB-N content reaches the national standard limit value, the average TVC has reached 6.38 (lg (CFU/g)), and the pork has been completely spoiled. Therefore, grading pork freshness only based on TVB-N content has certain limitations and is not enough to provide consumers with a safe and reliable reference [33,34]. In this study, the two key indicators of TVC and TVB-N were combined, and the freshness of pork was divided into two grades: fresh meat (TVC < 6 (lg (CFU/g)), TVB-N < 15 mg/100 g) and deteriorated meat (TVC > 6(lg (CFU/g)) or TVB-N > 15 mg/100 g). If the two key indicators of fresh meat meet the standards, it can be eaten with confidence. If either the TVC or TVB-N content level in deteriorated meat exceeds the standard, the pork has begun to corrupt, which poses a serious food safety risk. Meat of this kind should be eaten with caution or not at all. All samples were divided into a calibration set and a prediction set at a ratio of 3:1 by a random selection method. There were 72 samples in the calibration set and 24 samples in the prediction set. The two types of samples were designated 0 and 1, respectively, and the freshness of each subset of samples is shown in Table 1.

### 3.2. Hardware Testing

**Figure 4:**
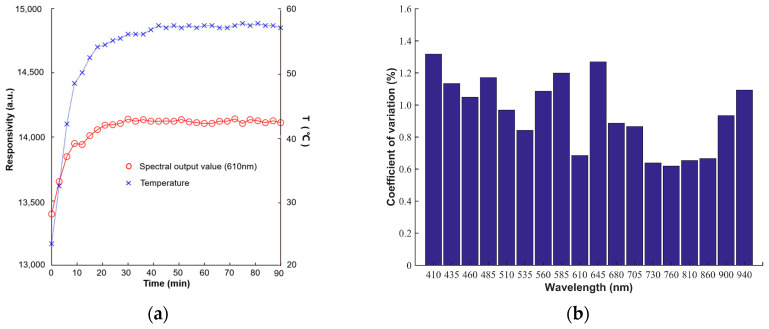
(**a**) Temperature and spectral output value of the multispectral system in 90 min; (**b**) coefficient of variation of 50 output values of multispectral system after preheating.

The stability of the temperature and spectral output of the multispectral system is extremely important for the detection accuracy of the detection device. In order to study the influence of warm-up time on system temperature and spectral output, the temperature and multi-spectral output value of the detection device were continuously monitored for 90 min after startup, and the detection interval was 3 min. Figure 4a shows the temperature and spectral output values (610 nm) as a function of the warm-up time. With the extension of preheating time, both the temperature and spectral output value increase. When the preheating time reaches 30 min, the temperature and spectral output of the device tend to be stable, the temperature is stable at about 57 °C, and the change trend of other wavelengths is the same as that of 610 nm. After the device was warmed up and stabilized, 50 white reference diffuse reflectance multispectra were collected continuously with the detection device. Figure 4b shows the coefficient of variation of 18 wavelengths of the detection device for 50 outputs, and the coefficient of variation of each wavelength is lower than 1.35%, indicating that the output response of the detection device has good stability when the preheating time is more than 30 min.

### 3.3. Diffuse Reflectance Multispectral Analysis

**Figure 5:**
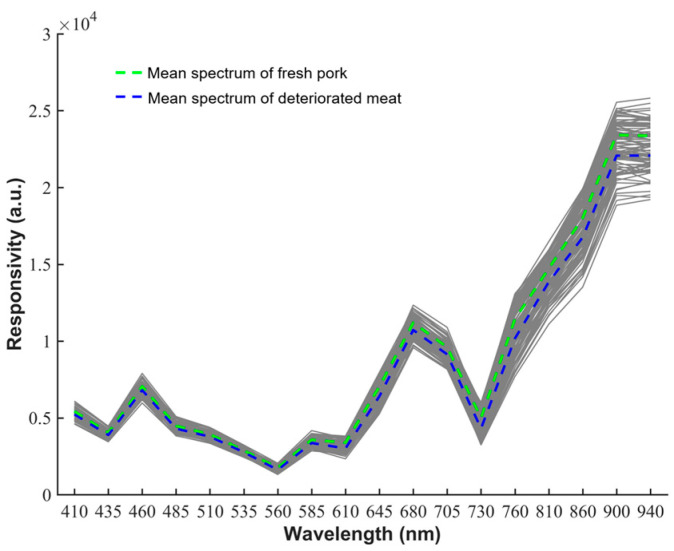
Diffuse reflectance multispectral (gray) of 96 pork samples with different freshness.

Multispectral variable analysis includes freshness discrimination based on a univariate analysis and freshness discriminant analysis based on multispectral shape features. The pork diffuse reflectance spectrum is classified according to different freshness levels, as shown in Figure 5, which shows the diffuse reflectance spectrum of all samples in the wavelength range of 410–940 nm (grey) and the average diffuse reflectance spectrum of fresh meat (green) and deteriorated meat (blue). Compared with fresh meat, the multispectral intensity of diffuse reflection is generally lower in deteriorated meat at different wavelengths, especially in the near-infrared band. Two reductions in diffuse reflectance spectral intensity occur at 560 nm and 730 nm, respectively. This may be due to the absorption peak of oxidative hemoglobin in the band around 560 nm [35] and the absorption peak of O-H bonds three times frequency band around 730 nm [36].

### 3.4. Analysis Results for a Single Variable

**Figure 6:**
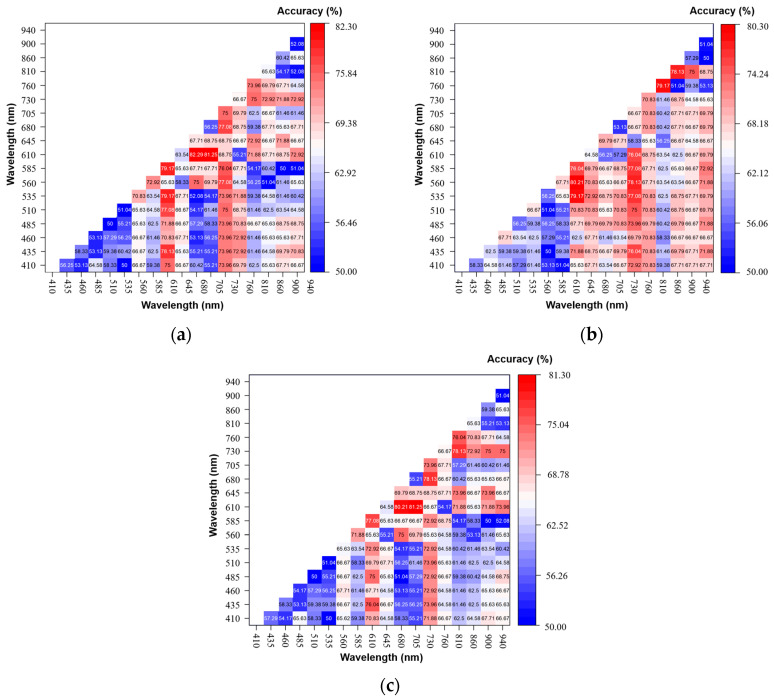
The discriminative effect of a single variable of spectral shape feature on pork freshness: (**a**) SR; (**b**) SD; (**c**) NSID.

Since most traditional spectral preprocessing methods usually need to process the entire continuous spectral band to obtain an ideal corrected spectrum, we can easily ignore the importance of a single variable, and thus it is not suitable for the correction of discrete spectral data. Therefore, more effective spectral information can be obtained by calculating the spectral morphological features of the multispectral spectrum, and the light scattering effect can be eliminated at the same time. A total of 153 SR variables, 153 SD variables, and 153 NSID variables were obtained. In this paper, the identification threshold of a single variable is determined by the Otsu method to verify the ability of a single spectral shape feature variable in the original multispectral data to discriminate the freshness of pork. The result of distinguishing pork freshness by a single spectral shape feature variable is shown in Figure 6.

Among the spectral ratios, the spectral indices calculated at 610 nm and 730 nm obtained the best discrimination results, with a classification accuracy of 82.29%, as shown in Figure 6a. Among the spectral differences, the spectral indices calculated at 560 nm and 610 nm obtained the best discrimination results, with a classification accuracy of 80.21%, as shown in Figure 6b. Among the normalized spectral intensity differences, the spectral indices calculated at 610 nm and 705 nm obtained the best discrimination results, with a classification accuracy of 81.25%, as shown in Figure 6c. At the same time, it can be seen from the results of the three kinds of spectral shape feature variables for discriminating pork freshness that the spectral indices with higher classification accuracy are all related to the wavelength of 560 nm, 610 nm, 705 nm, 730 nm, and 760 nm. This may be because the band around 560 nm is the absorption peak of oxidative hemoglobin, 610 nm is in the absorption area of the S-H bond thiomyoglobin in pork [4,10], and 705 nm, 730 nm, and 760 nm are in the band where the absorption peak of the O-H bond triple frequency doubles [34,35]. These wavelengths are all related to the spoilage of pork. Nevertheless, the highest discrimination accuracy of pork freshness based on a single variable of spectral shape feature is only 82.29%, which is insufficient to meet the needs of pork freshness evaluation.

### 3.5. Analysis Results of Different Shape Features and Their Combinations

In this study, partial least squares discriminant analysis (PLS-DA) was used to discuss the ability of different spectral shape features and their combinations to discriminate pork with different freshness levels, and the discriminative model of pork freshness was established according to the best spectral shape characteristic variable. Table 2 shows the discrimination results of pork freshness via the PLS-DA modeling method based on different spectral shape features and their combinations. The original multispectral modeling can achieve better prediction results than the univariate modeling, with an accuracy of 87.50% for freshness discrimination in the calibration set and 83.33% in the prediction set. Compared with the original multispectral modeling, the performance of the model after the preprocessing of the spectral shape features is significantly improved. Among them, the discriminant model established after SR and NSID preprocessing has better prediction accuracy, and the model performance is similar. The freshness discrimination accuracy of the correction set is 93.06%, and the freshness discrimination accuracy of the prediction set is 87.50%. However, when the three spectral shape features are combined to establish the discriminant model, the discriminant accuracies of the correction set and the prediction set are not significantly improved, and even the variability and accuracy of the calibration set are reduced. This may be because the three spectral shape features are used in combination, with a total of 459 variables. From Section 3.4, it can be seen that most of the variables have poor discriminant results and belong to non-informative variables. An increase in non-informative variables reduces the predictive ability of the PLS-DA model [28,37]. Therefore, in order to obtain a concise and stable discriminative model, which is convenient for loading and using the handheld detection device, it is necessary to screen the spectral shape features.

### 3.6. Variable Selection

The existence of a large amount of redundant spectral data will not only reduce the prediction accuracy of the model, but will also increase the computational burden and reduce the detection speed. To eliminate invalid variables, the most influential variables were selected from the PLS-DA model developed based on three spectral shape features (SR, SD, NSID). The weighted regression coefficient is considered to be the most sensitive indication of wavelength and accounts for most of the variation in the corresponding analysis [36,37,38].

**Figure 7:**
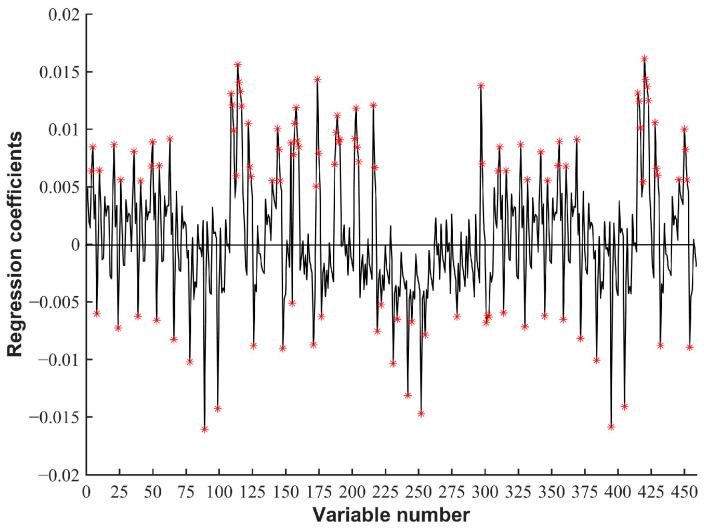
Regression coefficients of PLS-DA model based on combination of spectral characteristic variables.

Figure 7 shows that there are 109 spectral characteristic variables that are significantly affected by the regression model. The variable serial number is composed of three shape feature variables, SR, SD, and NSID, from bottom to top and from left to right (as shown in Figure 6). The selected feature variables are highly coincident with the red-colored spectral features in Figure 6. For example, the 110th and 111th variables correspond to 610/680 and 610/705, respectively. The 230th variable corresponds to 560 − 610; the 416th and 417th correspond to (610 − 680)/(610 + 680) and (610 − 705)/(610 + 705), respectively. This indicates that the selected spectral characteristic variables contain effective spectral information that can fully reflect the spectral differences between fresh meat and deteriorated meat. A PLS-DA model was established for analysis using 109 selected spectral features (the red mark). As is shown in Table 2, compared with using single spectral characteristic features for discrimination, the model based on the preferred variable has better performance, and the sensitivity and accuracy of the validation set are improved, reaching 92.86% and 91.67%, respectively. Compared with the combination of all spectral shape features, the model based on the preferred variable has the advantages of fewer variables and higher discrimination accuracy. Therefore, in this study we decided to use the optimal 109 spectral shape features to establish the discrimination model of pork freshness, which is conducive to the realization of the rapid nondestructive testing of pork freshness in situ.

### 3.7. External Verification

**Figure 8:**
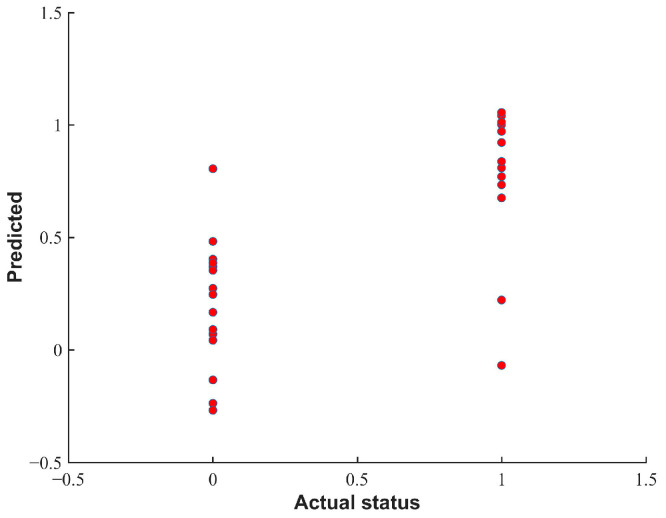
Discriminant results of pork freshness using PLS-DA model.

In order to test the accuracy of the freshness discrimination of the detector, the PLA-DA model established after variable selection was introduced into the device. Pork samples with different freshness levels were prepared according to the method in Section 2.1 for the validation of the device model, and 14 fresh meat samples and 16 deteriorated meat samples were obtained, respectively. As is shown in Figure 8, the discriminant accuracy of the validation set was 86.67%, and the sensitivity and specificity were 87.50% and 85.71%, respectively. The results showed that the detection device was accurate and reliable and could achieve the nondestructive and low-cost rapid screening of pork freshness.

## 4. Discussion

Many aspects of fresh pork production and marketing need to be carried out under cold storage conditions, and the narrow, closed, and humid testing environment limits the application of many technologies. At the same time, the test results of pork freshness have strong timelines, so nondestructive and rapid testing requirements are put forward for testing equipment technology. Previous studies have demonstrated that continuous wavelength visible/near-infrared spectroscopy can achieve the rapid nondestructive testing of pork freshness, but its high price and high processor requirements limit its field application [9,10,25]. In order to overcome these limitations, a real-time intelligent pork freshness detector based on multispectral technology was developed.

Compared with the traditional visible/near-infrared spectroscopy technology, the multi-channel spectral sensor technology has a limited number of wavelengths and a low separation rate. This limits its application in multi-quality inspection and precision quantitative inspection [24]. However, it has the advantages of low cost, small volume, and easy development in qualitative detection. In addition, spectral shape features can be used to calculate the shape information contained in spectral curves, eliminating the adverse effects of physical and biological variability on spectral information so as to improve the detection accuracy of multispectral sensors [21,25]. In this study, a multi-channel spectral sensor was combined with spectral shape features to realize the discrimination of pork freshness. The accuracy of the optimal model prediction set established by selecting spectral shape feature variables was 91.67%, and its accuracy with the independent verification set was 86.67%. The designed detector has the advantages of low cost, convenience, and simplicity. With the development of multichannel spectral sensor technology, it will be widely studied and applied in more fields.

## 5. Conclusions

In this study, an economical and efficient handheld pork freshness detection device was designed based on multi-channel spectral sensors. According to the content of TVC and TVB-N, pork is divided into two grades: fresh meat and deteriorated meat. The ability of the PLS-DA model to distinguish pork freshness based on different spectral shape features was compared. Compared with using the original spectrum directly, the spectral ratio, spectral difference, normalized spectral intensity difference, and the combination of different spectral types can obtain higher discriminant accuracy, but there are many redundant variables. According to the weighted regression coefficients of the PLS-DA model developed by three spectral shape feature combinations (SD, SR, and NSID), 109 spectral shape features were selected to establish an optimized PLS-DA model. Its accuracy with the prediction set is 91.67%. Its discriminant accuracy with the validation set is 86.67%, and its sensitivity and specificity are 87.5% and 85.71%, respectively. The device has good stability, is a reliable discriminant model, and can complete the detection in five seconds, which is conducive to the rapid and non-destructive screening of fresh pork during production, transportation, storage, and sale.

## Figures and Tables

**Table 1:** Class assignments of pork and their distributions in calibration and prediction sets.

Project	Deteriorated Meat	Fresh Meat
Class assignment	0	1
Calibration set	43	29
Prediction set	14	10

**Table 2:** Discrimination results of pork freshness by PLS-DA modeling methods with different spectral characteristics and their combinations.

Preprocessing Method	Number of Variables	Calibration Set			Prediction Set		
		Sensitivity (%)	Specificity (%)	Accuracy (%)	Sensitivity (%)	Specificity (%)	Accuracy (%)
NONE	18	86.05	89.66	87.5	78.57	90.00	83.33
SR	153	93.02	93.10	93.06	85.71	90.00	87.50
SD	153	90.70	93.10	91.67	78.57	90.00	83.33
NSID	153	93.02	93.10	93.06	85.71	90.00	87.50
SR&SD&NSID	459	86.05	93.10	88.89	85.71	90.00	87.50
VARIABLE SELECTION	109	93.02	86.21	91.67	92.86	90.00	91.67

## Data Availability

Not applicable.
